# Attachment anxiety is associated with a fear of becoming fat, which is mediated by binge eating

**DOI:** 10.7717/peerj.3034

**Published:** 2017-03-08

**Authors:** Katherine E. Alexander

**Affiliations:** Department of Psychology, College of Mount St. Vincent, Bronx, NY, United States

**Keywords:** Attachment theory, Fear of becoming fat, Eating behavior, Attachment anxiety, Binge eating

## Abstract

**Background:**

Previous work demonstrated that individuals with higher levels of attachment anxiety are prone to increased binge eating (Alexander & Siegel, 2013). Given that our society rejects obese individuals and individuals with higher levels of attachment anxiety tend to be highly sensitive to rejection (Downey & Feldman, 1996), it follows that those with increased attachment anxiety may be especially fearful of becoming fat.

**Methods:**

Undergraduate psychology students (*n* = 148) completed surveys measuring attachment, binge eating, and fear of becoming fat.

**Results:**

The data demonstrate that attachment anxiety is positively associated with a fear of becoming fat (*β* = .30, *p* < .001) and binge eating mediates this relationship. In other words, binge eating underlies the fear of becoming fat.

**Discussion:**

These findings contribute to a more refined understanding of binge eating which may create pathways for professionals to develop targeted interventions.

## Introduction

We live in a society simultaneously consumed with desires for unrealistic levels of thinness, and yet, suffers from an alarmingly high prevalence of obesity (Hill, Catenacci & Wyatt, 2005; Ogden et al., 2014). Conflicting societal messages both persuade individuals to eat more (and unhealthier food) while also remaining impossibly thin. Although most people will experience some form of these pressures, obese individuals frequently endure rejection from society via prejudice and discrimination (Burmeister & Carels, 2014; Crandall, 1994; King et al., 2006), potentially with very real consequences including whether they are hired for work (Agerstrom & Rooth, 2011).

Some individuals may be particularly susceptible to these pressures. Researchers have recently begun exploring individual differences in eating behavior through the lens of attachment theory (Alexander & Siegel, 2013; Troisi & Gabriel, 2011; Wilkinson et al., 2010). Attachment theory is a social/emotional construct researchers can use to model the way that individuals relate to their social and emotional environments (Hazan & Shaver, 1987). Attachment styles form within the first year of life and generally remain stable throughout the lifespan (Bowlby, 1988; Fraley et al., 2011; Scharfe & Bartholomew, 1994).

Attachment refers to the ways in which individuals form and maintain close social and emotional bonds, and researchers typically conceptualize patterns of attachment as either relatively “secure” or relatively “insecure” (Scharfe & Bartholomew, 1994; Noller & Feeney, 1996). Attachment security results from responsive and sensitive parenting, whereas attachment insecurity is the outcome of inconsistent or neglectful parenting (Bowlby, 1982; Bowlby, 1988). Attachment insecurity is predictive of eating disorders, decreased wellbeing and adjustment, and other maladaptive and risky behaviors (Cooper, Shaver & Collins, 1998; Greenberg, Siegel & Leitch, 1983; Lopez, Mitchell & Gormley, 2002; O’Shaughnessy & Dallos, 2009).

There are two dimensions of attachment insecurity: anxiety and avoidance (Mikulincer, Shaver & Pereg, 2003). Attachment anxiety is associated with increased fears of rejection and abandonment, negative views of self, and positive views of others (Bartholomew & Horowitz, 1991). The current study focuses on attachment anxiety, which is associated with increased binge eating (Alexander & Siegel, 2013). Increased attachment anxiety is also positively related to sensitivity to social rejection (Bartholomew & Horowitz, 1991; Downey & Feldman, 1996). Individuals with higher levels of attachment anxiety who become overweight are likely to suffer precisely the kinds of social rejection they fear due to pervasive anti-fat attitudes in society.

Unfortunately, individuals with higher levels of attachment anxiety are also more likely to engage in unhealthy eating behaviors, such as binge eating (Alexander & Siegel, 2013). Binge eating is a serious problem that is associated with obesity and other psychological problems (Grucza, Przybeck & Cloninger, 2007). About 70% of individuals with binge eating disorder are obese (Grucza, Przybeck & Cloninger, 2007). Binge eating disorder has recently been included in the fifth revision of the Diagnostic and Statistical Manual of Mental Disorders (DSM-5) (Call, Walsh & Attia, 2013). Overweight individuals who are known (by others) to binge eat are perceived as less attractive and blamed to a greater degree for their weight than obese persons who do not binge eat (Bannon et al., 2009), so binge eating may make those with increased attachment anxiety even more susceptible to rejection. Since anxiously attached individuals are more likely to binge eat, likely to be stigmatized if overweight, and likely to feel particularly hurt when stigmatized for being overweight, it follows that they would be especially fearful of becoming overweight.

The current study explores this pattern, first by replicating the association between attachment anxiety and binge eating found by Alexander & Siegel (2013), then by extending these findings by investigating the role of fear of becoming fat. Given the above, it seems likely that increased attachment anxiety would be associated with increased fears of becoming fat. Although no relationship between attachment anxiety and fear of becoming fat has been demonstrated, attachment anxiety is related to binge eating which in turn is related to concerns about weight (Ricca et al., 2009). Note that in this context, binge eating might be expected to mediate the fear of becoming fat: the increased tendency to binge eat among individuals with higher levels of attachment anxiety is likely to result in worries that the binge eating will lead to a gain in weight and the stigmatization that would follow. This amplifies the fear of social rejection. I therefore also predict that high levels attachment anxiety will be related to binge eating and fear of becoming fat, and that binge eating will mediate the relationship between attachment anxiety and fear of becoming fat.

## Materials & Methods

### Participants

148 undergraduate students (19 male) ranging in age from 17–31 (*M* = 19.3, SD = 1.52) participated in the study for class credit. Participants identified with the following ethnic groups: White (31.1%), Black/African American (11.5%), Asian (7.4%), Hispanic/Latino (48.6%), and Other/Mixed (1.4%). BMI ranged from 13.9–54.9 (*M* = 26.2, SD = 6.67). Three participants did not wish to be weighed so BMI could not be calculated for these participants. The study was approved by the Institutional Review Board at The College of Mount Saint Vincent.

### Procedure

Participants were asked to read and sign an informed consent. The consent form stated that the purpose of the study was to examine the relationship between eating behavior, weight, close relationships, and attitudes towards weight. Participants then completed a survey packet. At the end of the study, height and weight was measured. Finally, participants were debriefed. Participants were tested individually. The variables were measured using the:

1. The Experiences in Close Relationships-Revised (ECR-r; (Fraley, Waller & Brennan, 2000), a 36 item questionnaire measuring two dimensions of attachment insecurity: anxiety and avoidance. Scores range from 1 (low levels of insecurity) to 7 (high levels of insecurity). Over a three-week period, this measure demonstrates test-retest reliability scores of rs = .90 (Sibley, Fischer & Liu, 2005).
2. The Anti-fat Attitudes Questionnaire (Crandall, 1994), which contains three subscales measuring negative attitudes towards overweight individuals: dislike, willpower, and fear of fat. The entire questionnaire was given to participants, but the current analysis utilized the 3 item “fear of fat” subscale (*α* = .79). Scores range from 0 (low levels of fear) to 9 (high levels of fear).
3. The Binge Eating Scale (BES; Gormally et al., 1982), a 16 item measure of binge eating tendencies. Scores range from 0 (no binge eating problems) to 48 (severe binge eating problems). Timmerman (1999) found two-week test–retest reliability levels of *r* = .87.

Other questionnaires included for descriptive purposes:

1. The Rosenberg Self Esteem Scale (Rosenberg , 1965), a 10 item questionnaire measuring self-esteem (*α* = .88) (Robins, Hendin & Trzesniewski, 2001).
2. The Emotional Eating Scale (EES; Arnow, Kenardy & Agras, 1995) measures the desire to eat when experiencing anxiety (EES-Anx), depression (EES-Dep), and anger/frustration (EES-Ang). This scale demonstrates a two-week test-retest reliability of *r* = .79 (Arnow, Kenardy & Agras, 1995).
3. The differentiation of Self Inventory (DSI; Skowron & Friedlander, 1998) contains two subscales that can be used to assess emotion regulation ability: the emotional reactivity scale (*α* = .83) and the emotional cutoff scale (*α* = .80). These subscales are related to the dimensions of attachment insecurity: anxiety and avoidance, respectively (Wei et  al., 2005).

The order of the questionnaires was presented as: DSI, ECR-R, Self Esteem, EES, BES, and anti-fat attitudes.

## Results

Descriptive statistics are reported in Table 1. All statistical analyses were conducted using IBM SPSS Statistics Version 22. There were no differences in the results when broken down by ethnicity or gender. The following variables were positively skewed: antifat dislike, binge eating, emotional eating while frustrated, emotional eating while anxious, and antifat willpower. To address these skewed distributions, log and square root transformations were applied.

**Table 1:** Descriptive statistics.

Variable	Mean	Standard deviation
Attachment anxiety	3.22	1.11
Attachment avoidance	3.08	1.20
Fear of becoming fat	5.62	2.91
Anti-fat willpower	.60	.19
Anti-fat dislike	.78	.65
Binge eating	3.22	1.30
Self esteem	23.4	1.75
Emotional eating—anxiety	1.02	.34
Emotional eating—depression	1.82	.83
Emotional eating—anger/frustration	.97	.41
DSI—emotional reactivity	3.43	.81
DSI—emotional cutoff	4.23	.73

The data were checked for outliers. There were two outlying scores on the dislike variable and the emotional eating while angry variable. There were no errors in data entry and nothing atypical occurred during these participants’ sessions. Analyses were conducted with and without these participants’ data and the effects remained the same. Therefore, since the outliers were clearly not driving the effects there was no justification for removing them.

I predicted that individuals with increased attachment anxiety would experience an increased fear of becoming fat and binge eating would mediate the association between attachment anxiety and fear of becoming fat. Whereas a correlational analysis can only describe the relationship between variables, a mediational analysis can determine the underlying mechanisms of the relationship between two variables. All statistics for the mediation analysis were conducted using multiple regression and in coherence with steps outlined by Baron & Kenny (1986). According to this procedure, binge eating would be shown to mediate the link between attachment anxiety and fear of becoming fat if: (a) attachment anxiety is associated with binge eating, (b) attachment anxiety is associated with a fear of becoming fat, (c) binge eating is associated with a fear of becoming fat, and (d) when attachment anxiety and binge eating are entered into the model simultaneously, the statistical significance of the relationship between attachment anxiety and fear of becoming fat is diminished.

**Figure 1:**
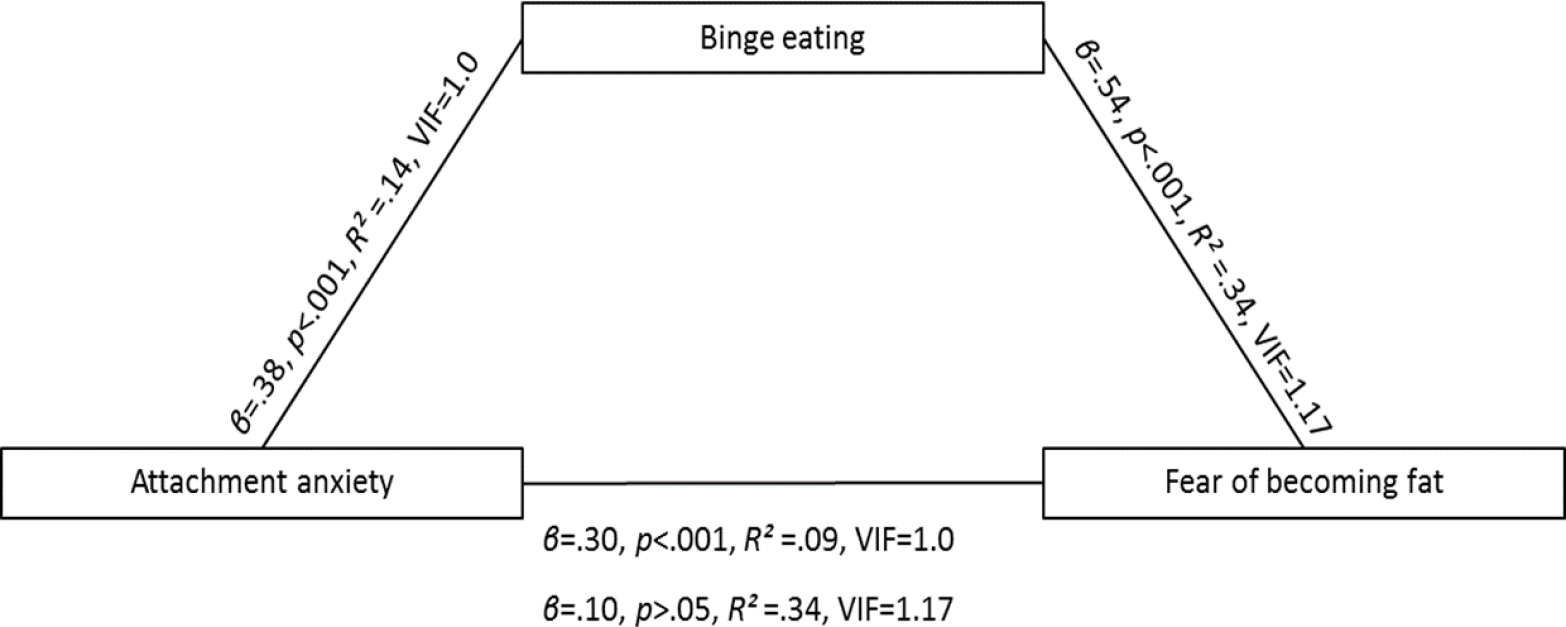
The relationship between attachment anxiety and fear of becoming fat is mediated by binge eating. Shown are the standardized *β*, *p*, and *R*^2^. The relationship between attachment anxiety and fear of becoming fat when binge eating is included in the model is shown in parentheses.

Correlations are reported in Table 2. In the first two steps, the predictor (attachment anxiety) was correlated with the outcome variable (fear of becoming fat) and the mediator (binge eating). In steps three and four, attachment anxiety and binge eating were both entered into the model. If mediation were present, the mediator (binge eating) would be correlated with the outcome variable (fear of becoming fat) and the relationship between the predictor (attachment anxiety) and the outcome variable (fear of becoming fat) would no longer be statistically significant. The criteria for each step of the mediation analysis were successfully met (see Fig. 1). The association between attachment anxiety and fear of becoming fat was mediated by binge eating. The Sobel test confirmed the mediation (*t* = 3.99, *p* < .000).

**Table 2:** Correlations.

	Attachment anxiety	Binge eating	Anti-fat fear	BMI
Attachment anxiety		.38^*^	.30^*^	.11
Binge eating			.58^*^	.45^*^
Anti-fat fear				.35^*^
BMI				

**Notes.**

* *p* < .01 (2-tailed).

**Figure 2:**
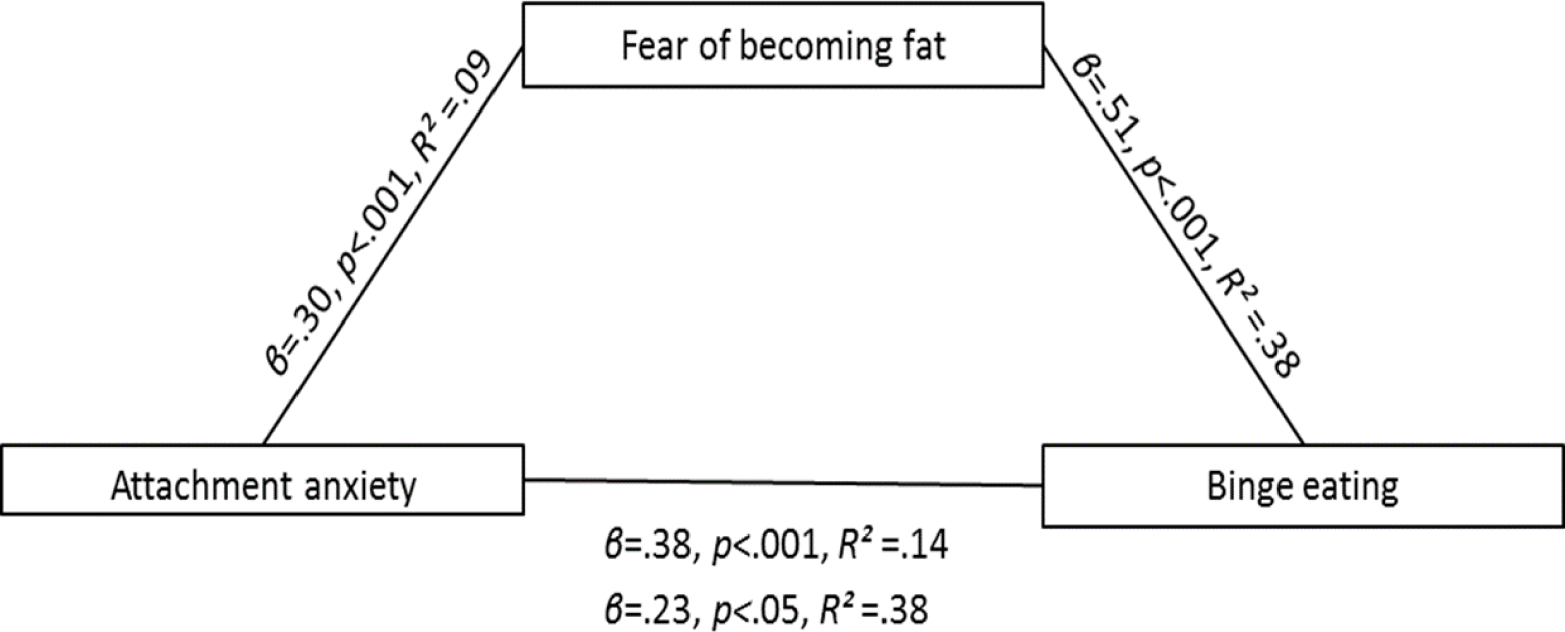
The relationship between attachment anxiety and binge eating is not mediated by fear of becoming fat. Shown are the standardized *β*, *p*, and *R*^2^.

Given that eating is an emotional experience, there was a possibility that the tendency for individuals with higher levels of attachment anxiety to binge eat might be influenced by their fear of becoming fat. To address this, a mediation analysis was also run to test whether the relationship between attachment anxiety and binge eating was mediated by fear of becoming fat. Step four of the mediation criteria was not met (see Fig. 2). The relationship between attachment anxiety and binge eating remained statistically significant when fear of becoming fat was entered into the model (*β* = .23, *p* < .05, *R*^2^ = .38).

Thus, individuals with higher levels of attachment anxiety are more likely to fear becoming fat because they are more likely to binge eat. However, these individuals with higher levels of attachment anxiety do not binge eating because they fear becoming fat.

## Discussion

Individuals with higher levels of attachment anxiety are susceptible to numerous maladaptive behaviors, including unhealthy eating behaviors (Alexander & Siegel, 2013; Troisi & Gabriel, 2011; Wilkinson et al., 2010). The current study found that individuals with increased levels of attachment anxiety experience an elevated fear of becoming fat. This study also illuminated the underlying reason for this relationship: binge eating. While it is not surprising that binge eating would be related to a fear of becoming fat, the relationship of attachment to eating behavior and weight is understudied and to my knowledge this is the first reporting of an association between attachment anxiety and a fear of becoming fat. Interestingly, as the current results demonstrate, attachment anxiety is not correlated with BMI itself (see Table 2 for correlations). Attachment anxiety is related to binge eating and fear of becoming fat (both of which are positively correlated with BMI). Therefore, the role that attachment anxiety could possibly play in obesity is not a direct path. Despite the fact that those with higher levels of attachment anxiety experience a greater fear of becoming fat, they are not actually more likely to be overweight.

I also replicate previous work demonstrating that attachment anxiety is associated with self-reported binge eating tendencies (Alexander & Siegel, 2013). These two findings are worrisome. The evidence of prejudice, discrimination, and overall stigma towards obese individuals is well documented (Burmeister & Carels, 2014; Crandall, 1994; Carr & Friedman, 2005; Puhl & Brownell, 2001; Crocker, Cornwell & Major, 1993; Agerstrom & Rooth, 2011). For example, customer service workers use more language with negative affect and are ruder to obese customers than customers of average weight (King et al., 2006). Obese individuals are 40%–50% more likely to report work related and interpersonal discrimination (Carr & Friedman, 2005). In a study by Klassen, Jasper & Harris (1993), participants who read fictitious employee summary sheets stated a preference to work with thin employees over obese employees. Benzeval, Green & Macintyre (2013) found that obese individuals are more likely to earn lower wages than their thin counterparts. Crocker, Cornwell & Major (1993) gave participants negative feedback from a fictitious dating scenario and overweight participants were more likely to believe the negative nature of the feedback was due to their weight than average weight participants. In short, overweight individuals are frequently rejected. Insensitive and unresponsive parenting experienced as infants leads to an increased sensitivity to rejection among adults with higher levels of attachment anxiety (Bowlby, 1988; Downey & Feldman, 1996; Mikulincer, Shaver & Pereg, 2003). The fear of becoming fat demonstrates the pervasive and generalized fear of rejection that those with attachment anxiety experience. These individuals may be more susceptible to the explicit societal pro-thin messages that surround us.

The current work further suggests that binge eating is the underlying reason that individuals with higher levels of attachment anxiety fear becoming fat. Importantly, the relationship between attachment anxiety and binge eating is not mediated by a fear of becoming fat. Individuals with increased attachment anxiety do not binge eating because they fear becoming fat, but rather, fear becoming fat because they binge eat. Individuals with higher levels of attachment anxiety may recognize that binge eating is associated with weight gain. Therefore, even though those with higher levels of attachment anxiety are not more likely to have a higher BMI, they may be self-aware that they engage in behaviors that could lead to becoming overweight and this provokes fear of becoming fat.

These fears may not be completely irrational. Bannon et al. (2009) found that obese individuals who binge eat were more stigmatized than those who do not. Thus, it is logical that individuals with higher levels of attachment anxiety who are already sensitive to rejection would be especially fearful of gaining weight.

We are surrounded with messages that being overweight is unacceptable. To some extent, it is “normal” to think negatively about being overweight. However, underlying attachment anxiety is a maladaptive emotion regulation strategy. Individuals who report higher levels of attachment anxiety tend to be emotionally reactive, i.e., extreme emotional responses to situations, hypersensitivity in emotional expression (Mikulincer, Shaver & Pereg, 2003; Wei et al., 2005). Additionally, there is evidence that binge eating is positively associated with maladaptive emotion regulation strategies (Czaja, Rief & Hilbert, 2009). Therefore, those with increased attachment anxiety are less equipped to handle a fear of becoming fat in a healthy way.

The recent inclusion of binge eating disorder to the DSM 5 warrants continuing research on the topic to help understand those experiencing the problem. Grucza, Przybeck & Cloninger (2007) state that binge eating disorder may be more common than other eating disorders. In a community sample, about 6.6% of individuals were labeled with binge eating disorder and about 70% of individuals with binge eating disorder were obese (Grucza, Przybeck & Cloninger, 2007). While obesity itself is a serious public health problem, when combined with binge eating disorder, it becomes even more problematic. Obese individuals who also exhibit binge eating are stigmatized to a greater degree than those who are only obese (Bannon et al., 2009). Obese individuals who also meet the criteria for binge eating disorder experience more psychiatric issues (Grucza, Przybeck & Cloninger, 2007) and have less success in weight loss reduction (Agras et al., 1997). Given the seriousness of binge eating disorder as a public health issue and on an individual level, the findings of this study are useful in contributing more knowledge about binge eating to the field.

The study had some limitations that are worth noting. The questionnaires were not counterbalanced. The only concern with this would be that priming participants to reflect on their eating behavior might have made their fear of becoming fat attitude more accessible, cognitively. However, even if their fear of becoming fat was magnified, it probably would not prime a response that did not exist. Additionally, although the sample was ethnically diverse, there were few males. However, when the data from male participants were removed from the sample, the results were not significantly different. Therefore, the data from male participants were included in the analysis.

## Conclusions

Fears of weight gain and binge eating are complex issues and many factors play a role in the manifestation of these problems, but, based on the current work, these issues are especially relevant to those with increased attachment anxiety. This study offers a distinct viewpoint by connecting the issue to attachment and fear of becoming fat. Additionally, attachment anxiety alone does not indicate a greater risk of becoming overweight, despite the fact that individuals with increased attachment anxiety are more likely to fear becoming fat. Although speculative, based on these findings, when working with individuals with elevated attachment anxiety, professionals may find it more effective to assess for and target binge eating tendencies in order to alleviate fears of becoming fat, rather than targeting fears of becoming fat directly. Future work should test relevant interventions. Professionals who work with individuals with eating issues should be aware that those with increased attachment anxiety may be more likely to engage in unhealthy eating habits and harbor more intense fears concerning those habits.

##  Supplemental Information

10.7717/peerj.3034/supp-1 Supplemental Information 1 Raw data

## Additional Information and Declarations

### Data Availability

The following information was supplied regarding data availability: The raw data has been supplied as a Supplementary File.
